# Structure-Function Insights of Jaburetox and Soyuretox: Novel Intrinsically Disordered Polypeptides Derived from Plant Ureases

**DOI:** 10.3390/molecules25225338

**Published:** 2020-11-16

**Authors:** Matheus V. Coste Grahl, Fernanda Cortez Lopes, Anne H. Souza Martinelli, Celia R. Carlini, Leonardo L. Fruttero

**Affiliations:** 1 Graduate Program in Medicine and Health Sciences, Brain Institute of Rio Grande do Sul (InsCer), Pontifícia Universidade Católica do Rio Grande do Sul (PUCRS), Porto Alegre CEP 90610-000, Brazil; matheusgrahl@hotmail.com; 2 Graduate Program in Cellular and Molecular Biology, Center of Biotechnology, Universidade Federal do Rio Grande do Sul (UFRGS), Av. Bento Gonçalves 9500, Building 43431, Porto Alegre CEP 91501-970, RS, Brazil; fernandacortezlopes@gmail.com; 3 Department of Biophysics & Deparment of Molecular Biology and Biotechnology-Biosciences Institute (IB), Universidade Federal do Rio Grande do Sul, UFRGS, Porto Alegre 91501-970, RS, Brazil; ahsmartinelli@yahoo.com.br; 4 Brain Institute and School of Medicine, Pontifícia Universidade Católica do Rio Grande do Sul (PUCRS), Porto Alegre 90610-000, RS, Brazil; 5 Departamento de Bioquímica Clínica, Facultad de Ciencias Químicas, Universidad Nacional de Córdoba, Córdoba CP 5000, Argentina; 6 Centro de Investigaciones en Bioquímica Clínica e Inmunología (CIBICI), Consejo Nacional de Investigaciones Científicas y Técnicas (CONICET), Córdoba CP 5000, Argentina

**Keywords:** biopesticides, antifungal activity, insecticidal activity, mechanism of action, transgenic crops

## Abstract

Intrinsically disordered proteins (IDPs) and intrinsically disordered regions (IDRs) do not have a stable 3D structure but still have important biological activities. Jaburetox is a recombinant peptide derived from the jack bean (Canavalia ensiformis) urease and presents entomotoxic and antimicrobial actions. The structure of Jaburetox was elucidated using nuclear magnetic resonance which reveals it is an IDP with small amounts of secondary structure. Different approaches have demonstrated that Jaburetox acquires certain folding upon interaction with lipid membranes, a characteristic commonly found in other IDPs and usually important for their biological functions. Soyuretox, a recombinant peptide derived from the soybean (*Glycine max*) ubiquitous urease and homologous to Jaburetox, was also characterized for its biological activities and structural properties. Soyuretox is also an IDP, presenting more secondary structure in comparison with Jaburetox and similar entomotoxic and fungitoxic effects. Moreover, Soyuretox was found to be nontoxic to zebra fish, while Jaburetox was innocuous to mice and rats. This profile of toxicity affecting detrimental species without damaging mammals or the environment qualified them to be used in biotechnological applications. Both peptides were employed to develop transgenic crops and these plants were active against insects and nematodes, unveiling their immense potentiality for field applications.

## 1. Introduction

Intrinsically disordered proteins (IDPs) and intrinsically disordered regions (IDRs) fail to form a stable tridimensional structure, challenging the century-old paradigm that a biological function is a specific property of a unique structure. In spite of the lack of an ordered structure, these proteins exhibit vital biological activities and can be found in all organisms, especially in eukaryotes [1,2]. IDPs and IDRs differentiate from structured proteins and domains, and they are characterized by notable conformational flexibility and structural plasticity. One of the differences of IDPs/IDRs and structured proteins include the amino acid composition since IDPs/IDRs are rich in disorder-promoting residues, such as Arg, Pro, Gln, Gly, Glu, Ser, Ala, and Lys. Also, IDPs/IDRs have low sequence complexity, high net charge, low mean hydropathy and are highly dynamic [2,3].

Many IDPs gain secondary structure when binding onto surfaces, for example, to a cell membrane [2]. Since IDPs/IDRs cannot fold spontaneously and some of them require partners to acquire a more ordered structure, these proteins do not have a code that defines the capacity of foldable proteins to fold spontaneously into a biologically active structure [4]. A typical IDP/IDR has a multitude of elements for potentially foldable, partially foldable, differently foldable, or unfoldable protein segments [5,6]. Their folding can be acquired after the interaction with proteins, nucleic acids, membranes, or small molecules. These conformation modifications can be driven also by changes in the IDPs environment as well as post-translational modifications. These IDPs can remain substantially disordered or become tightly folded after interaction [7,8,9].

IDPs are difficult proteins to study, due to their dynamic conformational landscapes changing between different structures on a variety of time scales [10,11]. Biophysical studies are crucial to clarify the relationship of the IDPs biological functions and their structures. Recent advances in heteronuclear multidimensional nuclear magnetic resonance (NMR) have allowed the complete assignment of resonances for several IDPs. NMR can also provide data about mobility of the unstructured regions [4,11]. In this way, NMR is possibly the most powerful technique for structural studies of these disordered proteins [12]. Furthermore, computational studies assumed an increasingly importance in interpreting these challenging experimental data [11].

## 2. Jaburetox and Soyuretox: Historical Aspects and Potential as Biopesticides

### 2.1. Transgenic Plants Expressing Biocide Polypeptides and Plant Defense

Hunger continues to afflict mainly the poorer countries around the world. In 2016, 10.7% of the world population were chronically undernourished (www.worldhunger.org). According to data provided by the World Bank (www.databank.worldbank.org), over the last 15 years, the word population has increased with an annual growth rate of ~1% (1.075 in 2019), from 6.59 (2006) to 7.67 (2019) billions inhabitants. With the increase in life expectancy, from 69.2 (2006) to 72.5 (2018) years, particularly in the richer countries, even considering a decrease of growth rate, estimates are that the world population could reach 9.7 billion (www.population.un.org) in 2050. By then, food demand will be 60% higher (www.webforum.org). As agricultural land is finite (it increased only from 47.18 in 2006 to 48.43 in 2016 millions square kilometers according to the World Bank), and is expected to shrink due to urbanization, climate change and soil degradation, increases in food production will require an even more efficient agriculture. The output of agriculture is hampered, however, by losses in the field or after harvesting, by a variety of insect pests, nematodes, fungi and diseases induced by bacteria or viruses [13,14]. Herbivores alone feeding on foliage, sap and root can decrease more than 20% of net plant productivity and food losses to insects are expected to even grow in a scenario of global warming [15].

To efficiently control insect pests in agriculture, combining different strategies is frequently required, including the use of resistant crop varieties. When there is no natural plant genotypes genetically resistant to insect pests, development of genetically modified (GM) resistant plants is an option. A milestone in the development of insect-resistant crops was established in the late 1980′s, by genetically engineering a tobacco plant to express an entomotoxic protein from the bacterium *Bacillus thuringiensis* (Bt) [16,17]. In 2018, the list of genetically modified plants that were commercialized had 26 species, either tolerant to herbicides or with increased resistance to insects (most expressing *Bt* toxins), grown or imported in 75 countries (www.gmoanswers.com). *Bt* toxins (or Cry proteins) have some restrictions, such as the low toxicity against sap-sucking insects [18,19]. Moreover, an increasing level of resistance of insects against Cry insecticidal proteins has been reported [20]. Fortunately, there is a number of plant entomotoxic proteins that can be used instead or in synergy with the *Bt* technology to control insect pests in new generations of transgenic plants yet to be developed [21].

### 2.2. Plant Proteins and Peptides with Insecticidal and Fungitoxic Properties

Insect pests and phytopathogenic fungi are detrimental to several crops and cause significant economic losses in agriculture worldwide. To cope with herbivory and fungal diseases, plants have evolved sophisticated defense mechanisms. Plant tissues accumulate, constitutively or after induction, various classes of defensive compounds that confer resistance against herbivores and infection by fungi, bacteria, viruses, as well as nematodes. The most known plant proteins involved in defense mechanisms against insect pests include lectins [22], ribosome-inactivating proteins of types 1 and 2 [23], inhibitors of proteolytic enzymes and glycohydrolases [24], modified forms of storage proteins [25,26], among others. Several plant peptides display antifungal properties such as defensins [27], lipid transport proteins [28], chitinases [29], lectins [30], thionins [31], cyclopeptide alkaloids [32] and other less common types. For a general review on these topics please refer to Dang and Van Damme [33] and Grossi-De-Sá et al. [21].

Ureases represent another group of plant proteins with insecticidal and antifungal properties which widen the proposed physiological roles of these enzymes [34,35,36,37]. Ureases (urea amidohydrolase; EC 3.5.1.5) are well conserved and nickel-dependent enzymes that catalyze urea hydrolysis into ammonia and carbon dioxide, synthesized by plants, fungi and bacteria [38,39,40,41]. Canatoxin is a less abundant urease isoform isolated from *Canavalia ensiformis* (jack bean) seeds [42,43]. Structurally similar to the seed’s major urease, both proteins display insecticidal and antifungal properties independent of their ureolytic activity [35,38,39]. Soybean (*Glycine max*) and pigeon pea (*Cajanus cajan*) ureases were also shown to display insecticidal [44,45,46] and antifungal [44,47,48] properties. Noteworthy, ureases are insecticidal against hemipteran pests (such as the stink bug *Nezara viridula* and the cotton stainer bug *Dysdercus peruvianus*), which were not susceptible to the entomotoxic activity of Cry toxins from *B. thuringiensis* [18,19]. Since these proteins are abundant in many edible vegetables, particularly in legumes, they can be generally regarded as biosafe [49].

### 2.3. Ureases and Derived Peptides as Sources of Insecticidal and Fungitoxic (Poly)Peptides

The insecticidal [50] and fungitoxic [51] effects of canatoxin were described before its characterization as an isoform of jack bean urease (JBU) [43]. In the first study of canatoxin’s insecticidal effect, it became clear that only insects relying on cathepsin-like digestive enzymes (such as the cowpea weevil *Callosobruchus maculatus* and the kissing bug *Rhodnius prolixus*) are sensitive to the toxin, while insects with digestion based on trypsin-like enzymes (such as the tobacco hornworn *Manduca sexta* or the fruitfly *Drosophila melanogaster*) show no susceptibility. The hypothesis of a proteolytic activation of the toxin was then proposed [50]. The hydrolysis of canatoxin with *C. maculatus* digestive enzymes yielded a 10 kDa entomotoxic peptide named pepcanatox [52]. Our group has demonstrated through inhibition of cathepsin-like enzymes, that the enzymatic activity of cathepsin B (cysteine proteinase) and cathepsin D (aspartic proteinases) is necessary for the release of toxic fragments of canatoxin [50,52]. Cathepsin B is a cysteine proteinase that can act as an exopeptidase or endopeptidase at acidic pH [53]. Cleavage by cathepsin B has a preference for basic and hydrophobic amino acids [54]. Meanwhile, cathepsin D cleavage occurs at acidic pH and has a preference for hydrophobic residues [55]. Subsequently, the major proteolytic activities of midgut homogenates of *D. peruvianus* nymphs were shown in vitro to catalyze the release of pepcanatox from JBU [56]. Cysteine, aspartic and metalloproteinases are present in both homogenates. Fluorogenic substrates containing JBU partial sequences flanking the N-terminal or the C-terminal portion of the entomotoxic peptide were efficiently cleaved by the *D. peruvianus* nymph midgut homogenates. Different classes of enzymes in the homogenates cleaved both substrates suggesting that in vivo the release of the entomotoxic peptide results from the concerted action of at least two different proteinases [56].

Jaburetox-2Ec, a recombinant peptide with 93 amino acids (~11 kDa) equivalent to pepcanatox, was produced heterologously in *Escherichia coli* from the corresponding sequence of the JBU isoform JBURE-II [57]. Here the term “peptide” is used solely to emphasize the fact that it is a fragment of a much larger protein regardless of its molecular mass. Later on, the peptide called simply Jaburetox was developed, with the same urease-derived sequence and the 6 His tail found in Jaburetox-2Ec, but lacking the V5 epitope present in the latter [58]. Both peptides, Jaburetox-2Ec and Jaburetox, display equivalent insecticidal activity, evidencing that the epitope V5 is not implied in their entomotoxicity [58]. 

Since one of the most well studied mechanisms of defense against insect pest is digestive enzyme inhibition, this possibility was explored by our group for ureases and derived peptides. As described by Carlini et al. [50] and Ferreira da-Silva et al. [52], canatoxin showed no inhibitory effect on the proteolytic (cathepsin B or D-like) or α-amylase activities. Moreover, the peptides derived from Canatoxin’s digestion with cathepsin-like enzymes, including pepcanatox, did not display either proteolytic or amylase inhibitory properties [59]. Although Jaburetox itself was not tested, taking into account its virtually identical sequence when compared to pepcanatox, it is safe to assume that Jaburetox has no inhibitory effects upon digestive enzymes.

Jaburetox is lethal to several insects susceptible to canatoxin (the cotton stainer bug *D. peruvianus*, the kissing bugs *R. prolixus* and *Triatoma infestans*) and also kills insects that are resistant to intact ureases, such as lepidopterans (fall armyworm *Spodoptora frugiperda*, cotton bollworm *Helicoverpa armigera*) and dipterans (*Aedes aegypti*) [57,60], because the hydrolysis of the protein to release the peptide is no longer required.

Concerning the antifungal property of ureases and derived peptides, the most abundant jack bean isoform, JBU, was shown to display antifungal properties against a panel of 16 phytopathogenic filamentous fungi species of 11 genera, blocking spore germination and/or mycelial growth, and inhibiting multiplication of yeasts at submicromolar concentrations [47,61]. Jaburetox also displayed antifungal properties against filamentous fungi and yeasts [61].

Antifungal effects were observed in vitro also for isoforms of soybean urease (SBU) [47,62]. The participation of ureases in plant defense against fungal diseases was demonstrated in urease-null soybean plants obtained by gene silencing [63]. Later, the peptide called Soyuretox, homologous to Jaburetox, but derived from the ubiquitous isoform of the soybean urease, was heterologously expressed in *E. coli*, characterized structurally and its entomotoxic and antifungal effects were demonstrated [64]. 

**Figure 1:**
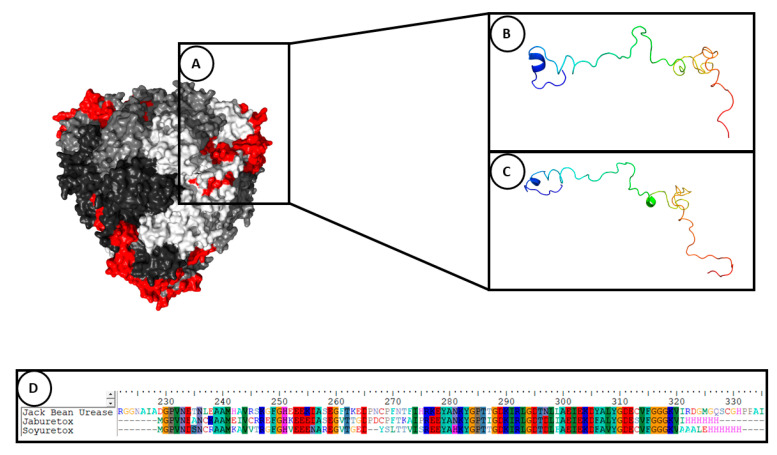
Structural representation of entomotoxic peptides and Jack Bean Urease. (**A**) Graphical representation of the location of the peptides (red) in the protein structure of Jack Bean Urease (pdb: 3LA4). Each monomer of the JBU hexamer is represented with a different shade of grey. (**B**) Jaburetox, (**C**) Soyuretox and (**D**) comparison of the primary sequences.

Jaburetox showed no acute toxicity to mice and rats [57] and was found not toxic in a risk assessment study [65] while Soyuretox was not toxic to zebrafish embryos [64]. These data suggest that these peptides may be safe alternatives to attain resistance to insect herbivory and/or fungal disease in transgenic plants. In the following sections, the structural aspects (Figure 1) and biological profile of Jaburetox and Soyuretox are reviewed.

## 3. Structural Aspects of Jaburetox and Soyuretox and Its Interaction with Membranes

Since the discovery of the insecticidal effect of Jaburetox, our group carried out different approaches aimed to identify the active region of the molecule and characterize the structure involved in this toxicity. In this direction, a first modeling for Jaburetox was performed employing an ab initio approach. Using this strategy, 10 different models of possible conformations of the peptide were proposed. In all of them, a β-hairpin motif appeared in the C-terminal region of the molecule, similar to those found in pore-forming peptides. Based on this, it was hypothesized that the β-hairpin motif could be involved in the entomotoxic activity of Jaburetox [57].

Two years later, molecular aspects of Jaburetox and its capability to interact with model membranes were demonstrated for the first time. In this study, Jaburetox displayed the ability to interact with and disrupt large unilamellar vesicles (LUVs) composed mainly by acidic lipids. Moreover, the interaction with LUVs were found to depend on the aggregation state of the polypeptide [66]. Dynamic light scattering and small angle X-ray scattering techniques were used to demonstrate the Jaburetox-lipid interaction with platelet-like multilamellar liposomes (PML) [67]. A tridimensional model indicated that Jaburetox could anchor at a polar/non polar lipid interface, and it was suggested that the β-hairpin motif could be the responsible for this membrane-disturbing effect [66]. The presence of the Jaburetox’s β-hairpin motif in the structure of JBU was confirmed by crystallography by the Ponnuraj group in India, who elucidated the 3D structure of JBU [68].

In order to identify the regions of the molecule involved in the biological activities of Jaburetox, truncated versions of the peptide were obtained by site-directed mutagenesis [58]. Three mutated versions were constructed and overexpressed in *E. coli*: (1) a peptide lacking the β-hairpin motif (residues 61–74 was deleted, the resulting peptide was called jbtx Δβ-hairpin); (2) a peptide corresponding to the N-terminal portion (residues 1–44, called jbtx N-ter); and (3) a peptide corresponding to the C-terminal region (residue 45–93, called jbtx C-ter). All these peptides were tested for their insecticidal activity and the ability to interact with LUVs. The peptide jbtx Δβ-hairpin displayed the same insecticidal effect of the whole peptide, demonstrating that β-hairpin was not involved in this toxicity. The peptide jbtx N-ter, corresponding to the N-terminal half of Jaburetox, showed insecticidal effect comparable to that of the native peptide, while jbtx C-ter, representing the C-terminal half, was largely inactive on two different insect models [43]. On the other hand, both jbtx N-ter and jbtx C-ter were able to interact with LUVs, the C-terminal half being more effective. As the amphiphilic β-hairpin is present in the C-terminal domain, this motif could be in part involved in membrane interaction. In contrast, for the entomotoxic activity, the half peptide jbtx N-ter contains the most important domain, as observed in the experiments. Three dimensional models for Jaburetox were proposed using bioinformatics and the polypeptide and its mutants were submitted to a molecular dynamic simulation in aqueous system for 500 ns. The results suggested that the whole peptide becomes more unstructured, particularly at its N-terminal portion, and contained a few secondary structure elements with a major part of molecule in random coil conformation at the end of the 500 ns simulation. Interestingly, the β-hairpin structure was conserved in the C-terminal half. When the mutants were submitted to simulation, the jbtx N-ter peptide became totally unfolded while the jbtx C-ter showed a stabilization with β-sheet structures after 500 ns molecular dynamics [58].

Jaburetox and its truncated peptides were also studied using an electrophysiological approach to test their ability to form channels in planar lipids bilayers (PLBs). Two different membrane compositions, to produce neutral net charged and negative net charged lipid interfaces, were tested. All peptides, Jaburetox, jbtx N-ter, jbtx C-ter and jbtx Δβ-hairpin, were able to form channels in both types of bilayers, observed within 30 min after addition of 5–15 µg/mL of each peptide. All channels showed similar biophysical properties, being highly selective to cations, and displayed two conducting states: 7 pS–18 pS and 32 pS–79 pS (smaller channel and main channels, respectively). Similar to Jaburetox, jbtx N-ter was more active at negative voltages while the others did not show voltage dependence. Multiple levels of currents were observed during the experiments using high doses of peptides, suggesting the presence of several identical channels or simultaneous activity of oligomers, corroborating previously reported data on the tendency of Jaburetox and its truncated peptides to form aggregates and the fact that the peptides’ oligomerization state influences their biological effects [58,66].

**Figure 2:**
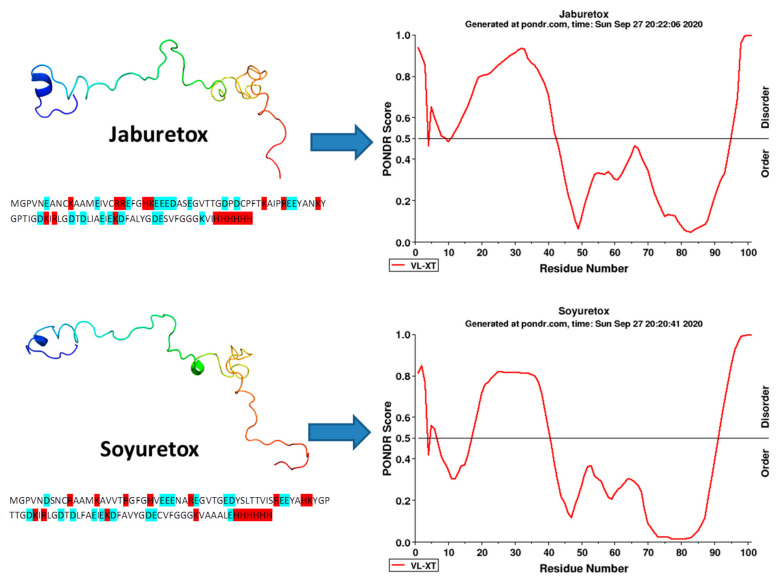
Disorder profile of Jaburetox and Soyuretox. Algorithm of disorder prediction VL-XT PONDR^®^ was applied to compare Jaburetox and Soyuretox amino acid sequences. The amino acid sequences (including the 6-His tags) were submitted to http://www.pondr.com/ to generate the graphics. PONDR score above 0.5 indicates disorder. Jaburetox is slightly more disordered than Soyuretox, especially in its N-terminal region. Jaburetox structure is based on conformer 1 (PDB 2MM8). The modeled structure of Soyuretox was obtained with Modeller 9.19 using Jaburetox as a model. The primary sequences of the peptides appear below each 3D-structure, negatively charged amino acids are highlighted in blue and positively charged in red.

A structural characterization of Jaburetox in solution was carried out using light scattering, circular dichroism (CD) spectroscopy and NMR, and demonstrated the intrinsically disordered nature of the polypeptide [69]. Light scattering studies of the hydrodynamics properties of Jaburetox showed that the peptide in a neutral solution is found in a single oligomeric form with a molar mass of 11.03 kDa. It exhibited a large hydrodynamic radius for a peptide of this molecular mass, a feature suggestive of a disordered polypeptide. CD spectroscopy revealed a typical random coil conformation of Jaburetox and no strong negative signals above 205 nm, thus indicating that Jaburetox presents small amounts of secondary structure. Computational analysis predicted a propensity to disorder mainly at the Jaburetox’s N-terminal domain (Figure 1). As Jaburetox tends to aggregate, a thermal scanning fluorimetric assay was performed to identify the best conditions to stabilize the peptide. In the presence of the reducing agent tris(2-carboxyethyl)phosphine (TCEP), the tendency of Jaburetox to aggregate was reduced allowing the NMR assays. The heteronuclear single quantum coherence (HSQC) spectrum unveiled low signal dispersion in the proton dimension which is an indicative of a disordered state of the polypeptide. The chemical shifts were used to predict the residue-specific propensity to form a secondary structure (SSP). SSP analysis predicted that Jaburetox is widely disordered with a small tendency to form α- structures, in addition, with slightly smaller SSP values in the N-terminal (larger predicted disorder) compared to the C-terminal portion (smaller predicted disorder), corroborating the disorder predictors (Figure 2). Data acquired from the nuclear Overhauser enhancement (NOE), which reflect the global fold state of the protein, did not demonstrate the presence of a stable tertiary structure. Still, some elements of secondary structure were detected in parts of the molecule, as a small α-helical motif in the N-terminal region (residues A12-V16), and two turn-like structures, one located in the middle of the polypeptide (residues R48-G56) and the other in its C-terminal region (residues I63-E74). However, the β-hairpin, evidenced in the JBU crystal and in the bioinformatics studies, was not observed in the 3D structure. An in-cell NMR spectroscopy was employed to investigate the molecular folding of the peptide in a physiological condition. A HSQC spectra recorded on *E. coli* cells overexpressing Jaburetox confirmed its disordered folding inside the cells [69].

In order to elucidate if Jaburetox could acquire some conformation when in contact with membranes, a study using artificial and biological membranes was performed [62]. When incubated with sodium dodecyl sulfate (SDS) micelles, a change in the secondary structure of Jaburetox was detected in its CD spectra. Moreover, NMR HSQC confirmed changes in its tertiary structure. Some conformational changes were observed in the molecule, mainly in its N-terminal region, when in contact with LUVs and micelles, prepared with different net charges and molar ratios of phospholipids. This effect was more visible with negatively charged LUVs and micelles, despite without major acquisition of tertiary structure, as determined by NMR. Fluorescence microscopy was employed to show the interaction between Jaburetox labelled with fluorescein isothiocyanate (Jaburetox-FITC) and cockroach’s nervous cord (NC) as a biological membrane, revealing a great intensity of Jaburetox-FITC attached to the ganglia. When Jaburetox-FITC was pre-incubated with LUVs and bicelles, before addition to insect NC membrane, the fluorescence decreased about 50% and 70%, respectively, compared to the initial values, suggesting that the vesicles competed with the insect membranes as a target for the binding of Jaburetox. These data provided evidence that the interaction between Jaburetox and phospholipids did not induce a complete transition from unfolded to folded state but it could have facilitated the peptide’s anchorage in cell membranes for posterior acquisition of a folded state [62].

The secondary and tertiary structures of Soyuretox were determined using bioinformatics tools, CD spectra and NMR experiments. Jaburetox was used in the same study for comparison. Both peptides share 68% of identity in their amino acid sequences (Figure 1). The secondary structure of Soyuretox and Jaburetox was analyzed by CD spectroscopy at pH 6.5 and 8.0. At pH 6.5 solution, both peptides maintained a disordered behavior. Nonetheless, while Jaburetox kept its disordered state at pH 8.0, Soyuretox acquired some secondary structure in the alkaline medium. A 3D model of Soyuretox was obtained using the structure of JBU as template and a molecular dynamics (MD) was carried out. After 500 ns of MD simulation, Soyuretox became more globular in solution and showed changes in its secondary structure, with loss of helices and beta strands [64]. In spite of the fact that Jaburetox and Soyuretox behave similarly, Soyuretox demonstrated a tendency to aggregate at pH 6.5 and 8.0 (at the protein concentrations necessary for NMR the experiment), preventing the assignment of the NMR signals [64,69]. The HSQC NMR spectrum obtained for Soyuretox showed a low signal dispersion in the proton dimension, typical of intrinsically disordered states. The ability of Soyuretox to interact with SDS micelles was studied by CD, revealing that in presence of 10 mM SDS (above critical micellar concentration) there was an increase in the peptide’s content of secondary structure. A more ordered structure of Soyuretox in the presence of SDS micelles (10 mM) was also confirmed in the peptide’s HSQC NMR spectrum, exhibiting a widening of signal dispersion. Under these conditions, Soyuretox kept its intrinsically disordered state [64]. A compilation of the main data obtained in the structural studies of Jaburetox and Soyuretox is present in Table 1.

## 4. Biological Studies

The basis of the apparent selectivity of Jaburetox and Soyuretox towards certain organisms without affecting others is not well understood. As mentioned before, studies of acute toxicity injecting or feeding Jaburetox in murine models resulted in no effect [57]. Standardized in vitro assays indicated that Jaburetox did not cause cito- or genotoxicity in human and other mammalian cell lines [70]. Regarding Soyuretox, embryotoxicity assays in the zebrafish model demonstrated the lack of effect of the peptide on several developmental and behavioral parameters [64]. On the other hand, both peptides presented potent insecticidal and antimicrobial effects (discussed in the following subsections). A possible explanation for this selectivity could be related to the fact that these peptides have more affinity for certain types of membrane lipids than others. Thus, the presence of these and possibly other molecules in susceptible cells could be necessary for the peptide to act upon. The biosecurity profile of Jaburetox seems promising since, in addition to the lack of toxic effects observed so far in vertebrates, a risk assessment study could not identify potential adverse reactions associated to the peptide [65]. Finally, the amino acid sequences of Jaburetox, Soyuretox and their homologs are present in ureases and these enzymes are abundant in several edible plants, including some that are eaten raw [71,72].

### 4.1. Entomotoxic Effects

The indiscriminate use of pesticides has caused a negative impact in the environment and in the health of consumers. Besides, it has facilitated the emergence of resistance in more than 600 insect and mite species [73,74]. Those phenomena have driven the study of new insecticidal molecules that, ideally, are environment-friendly and affect the target pest species without harming beneficial insects, humans and other animals [75]. According to the Insecticide Resistance Action Committee (IRAC), there are more than thirty types of modes of action currently described, including sodium channel modulators, juvenile hormone inhibitors and miscellaneous non-specific (multi-site) inhibitors, among others [73]. In the cases of Jaburetox and Soyuretox, their mechanism of action is still not completely understood. As discussed above, what we do know is that Jaburetox interacts with lipid membranes, especially those of acidic nature [66], and present cell membrane-disturbing activities [76]. We have reported that Jaburetox act upon several insect organs and at different levels, i.e., by altering the activity of various enzymes and/or the protein and the gene expression of several proteins [77,78]. The property of this peptide to target different organs, cell types, and proteins probably reflects its intrinsically disordered nature that would allow to accommodate the interaction of Jaburetox with different binding partners. Concerning Soyuretox, we know less about its sites of action, but as far as entomotoxicity goes, current evidence suggests that Soyuretox has properties similar to those of Jaburetox [64]. As commented before, Jaburetox and Soyuretox share 68% of their sequence whereas the N-terminal regions of the peptides are the most divergent. However, they can conserve functions and other features without necessarily presenting a conserved sequence as it has been described for different IDPs [79]. In an attempt to systematize and integrate the data obtained by our laboratory and collaborators regarding the entomotoxic effects of Jaburetox and Soyuretox, these findings were grouped hoping to throw light to some general trends, and trying to lay working hypothesis and new avenues for future research. The effects of Jaburetox and Soyuretox on different insect species are summarized in Table 2.

#### 4.1.1. Lethality

Like other insecticidal proteins, including the widely used Cry proteins of *B. thuringiensis* [89], the “parent” proteins of Jaburetox, canatoxin and JBU, or SBU, in the case of Soyuretox, need a step of proteolytic activation to act upon insects of different orders. Even though these urease isoforms present entomotoxic effects *per se*, they are not lethal when fed to insects with trypsin-based digestion [35]. This caveat can be surpassed by employing Jaburetox as first demonstrated by Mulinari et al. [57]. Since that finding, several species were tested for lethality and almost all of them were susceptible to Jaburetox. This peptide was effective via injection and oral administration against juveniles and adults. Moreover, the doses employed were very low when compared to other entomotoxic proteins derived from plants [35]. There are less information available for Soyuretox, although the effect of a dose comparable to those employed with Jaburetox also resulted lethal in *D. peruvianus* [64]. The only exception to this trend so far has been the cockroach *Nauphoeta cinerea*, since feeding or injecting Jaburetox did not result in mortality [86]. As we will discuss later, the effect on the activities of the central nervous system enzymes was different in cockroaches of this species when compared to susceptible insects such as the kissing bug *R. prolixus*. This difference could explain, at least in part, the resistant phenotype of *N. cinerea*.

#### 4.1.2. Effects on the Central Nervous and Neuromuscular Systems

Behavior alterations consistent with a neurotoxic activity of Jaburetox in *T. infestans* [84] led our group to further investigate the effects on the insect’s central nervous system. Immunohistochemical techniques evidenced the labelled somata of the antennal lobe and of the suboesophageal ganglion 3 h after the injection of the peptide into the hemocoel. Co-immunoprecipitation assays followed by tandem mass spectrometry identified the enzyme UDP-*N*-acetylglucosamine pyrophosphorylase as a Jaburetox-interacting protein in the bug’s brain and associated ganglia [84]. This enzyme provides the activated precursor for the synthesis of chitin and for glycosylation pathways of glycoproteins and other derived products [90].

The central nervous system was also a target for Jaburetox in the triatominae *R. prolixus*, and the interaction between the fluorescently-labeled peptide and the organ could be observed in vitro. Furthermore, the activities of different enzymes in the central nervous system were altered after feeding the peptide to fifth instar nymphs [78].

The interaction of Jaburetox with the nervous cord tissue of *N. cinerea* was observed in vitro [62]. Injection of the peptide into the cockroaches *Phoetalia pallida* and *N. cinerea* led to the blockade of evoked contractions of coxal muscle [58,85]. In *N. cinerea*, this effect was amplified by chloral hydrate, (a drug known to reinforce effects mediated by GABA receptors), suggesting that Jaburetox could be activating the gabaergic neurotransmission [85]. As reported in the kissing bugs, the enzymatic activities of several enzymes were modulated in adult cockroaches upon injection [86].

The effect of Jaburetox was tested on *Xenopus laevis* oocytes overexpressing the Nav 1.1 channels from the cockroach *Blatella germanica* [85]. Voltage-clamp analysis showed a 50% increase in the sodium currents upon Jaburetox treatment while no alteration in the kinetics of the Nav 1.1 channel activation was noticed. Muscle and nerve action potentials recorded in the isolated leg of the locust *Locusta migratoria* decreased transiently about 20% in Jaburetox-treated preparations, returning to basal values after 20 min although by then the contraction of the tarsus has stopped. The absence of a fast decrease in the resting membrane potential during the voltage clamp studies, especially considering the membrane-disturbing effects of Jaburetox, suggested that the main component of its neurotoxicity could involve alteration of the gating properties of sodium channels [85]. These aspects were recently reviewed by Barreto et al. [91].

#### 4.1.3. Effects on Behavior

Behavioral alterations were first reported in adult individuals of the triatomine *T. infestans* by Galvani et al. [84]. The injection of a lethal dose of Jaburetox that would eventually kill the insects within 18 h, led to early transient symptoms that included paralysis of the legs, proboscis extension and abnormal movements of the antennae. In fact, those findings pointed out to neurotoxicity, a phenomenon which was later on confirmed by diverse approaches and ascribed, at least in part, to alterations of the nitrinergic system in the central nervous system of this species [84]. In the case of the cockroach *N. cinerea* [85], the locomotor behavior was also altered after Jaburetox injection, and adult insects exhibited a significant decrease in the travelled distance accompanied by a corresponding increase in the stopping time. The leg and antenna grooming activities were also modified, with significant increments upon injection. These and other toxic effects were attributed to an initial activation of voltage-gated sodium channels [85].

#### 4.1.4. Effects on Enzymatic Pathways

Since the finding that the injection of Jaburetox diminished the enzyme activity of Nitric Oxide Synthase (NOS) in the central nervous system of *T. infestans* [84], a series of approaches were undertaken in order to try to understand the basis of this alteration. Besides its function in nitrinergic signaling in the central nervous system, NO participates in the immune response of insects due to its capacity of inducing oxidation of heme groups and nitrosylation of amino acid residues in proteins of pathogens [92]. The diminution of NOS activity upon Jaburetox injection was not related to the protein levels, since no differences in band intensities were seen in Western blots of brain homogenates of vehicle-injected and Jaburetox-injected insects [84]. The NOS activity also decreased when the homogenates were incubated with the peptide in vitro, suggesting a direct effect of the Jaburetox on the enzyme [84]. Similar results were obtained in the central nervous system of the related triatome *R. prolixus*, with both in vivo and in vitro Jaburetox treatments leading to a decrease in NOS enzyme activity without affecting its gene expression. Nevertheless, the effect of Jaburetox was different on the activity of NOS in the salivary glands and hemocytes, where the expression of its gene was increased, indicating an organ-specific effect [78]. Jaburetox-induced alterations of NOS is not restricted to triatomines, since the peptide also induced a decrease in NOS enzyme activity in the central nervous system of the cockroach *N. cinerea*, upon in vivo or in vitro treatments, without affecting the protein expression [86]. The regulation of NO production mediated by Jaburetox is complex and could involve more than one level, for example, affecting directly the enzyme as the in vitro assays pointed out and/or, indirectly, through modifications on the expression of its gene, or even altering the membrane properties of target cells [77].

Considering that Jaburetox interacted with UAP in the central nervous system of *T. infestans*, the effect of the peptide on this enzyme was also tested. It was observed that the UAP enzyme was affected either after in vivo or in vitro treatments with Jaburetox, in this case causing a significant increase in activity without modifying the expression of its corresponding gene. Again, an organ-specific effect was demonstrated, with different responses of UAP in the central nervous system as compared to the salivary glands and hemocytes [77]. When a recombinant version of the *R. prolixus*’ UAP was incubated in vitro with Jaburetox, no modification of the enzyme activity was observed, suggesting that other factor(s) present in the tissue homogenates are probably required for the peptide to exert its regulatory effect [78].

Taking into account the various functions of the main product of UAP, UDP-*N*-acetylglucosamine, several physiological processes can be influenced by the toxin. One of such process is chitin synthesis, catalyzed by chitin synthase and serving UDP-*N*-acetylglucosamine as substrate. The effect of Jaburetox on the expression of chitin synthase gene was then explored. It was found that Jaburetox treatment led to a diminution of chitin synthase expression in the central nervous system, salivary glands, anterior midgut, Malpighian tubules and fat body, but not in the hemocytes or the posterior midgut of *R. prolixus* [77,78]. The profile of UAP modulation by the Jaburetox in *N. cinerea* was different, since its activity was only affected 18 h after injection. This distinct response when compared to the triatomines could be related to the fact that this cockroach is so far the only species found to be resistant to the acute lethal effect of Jaburetox [86]. As the activity of acetylcholinesterase, an enzyme involved in the resistance of insects to organic pesticides, increased upon treatment of the cockroach with Jaburetox, the lack of lethality could be a reflex of an analogous mechanism(s) disabling the toxic effects of the peptide [86]. However, Jaburetox is far from being innocuous to *N. cinerea*, as the paralyzing effect and alterations in behavior caused by the peptide could lead to death as well, due to inability to hide and avoid danger, or to find food.

#### 4.1.5. Effects on Diuresis

The evaluation of the effects of Jaburetox on diuresis was one of the first investigations carried out to understand the mechanism of action of the toxin. In 2009, Stanisçuaski et al. [81] conducted studies on *R. prolixus*’ Malpighian tubules to explore, in vitro, the effects of Jaburetox on serotonin-induced diuresis. The authors demonstrated that Jaburetox and also JBU are capable of interacting with membrane factors that end up inhibiting diuresis by triggering different signaling cascades. While JBU effect is mediated by the activation of the eicosanoid cascade and is dependent on Ca^++^ ions, the antidiuretic effect of Jaburetox is mediated by an increase in cyclic guanosine monophosphate (cGMP) levels. This increment leads to the interruption of ion transport by blockage of the apical V-ATPase and disruption of the transepithelial potential across the tubule’s membrane through an unknown pathway, leading (directly or indirectly) to the inhibition of water secretion and consequent impairment of diuresis [81].

#### 4.1.6. Effects on the Immune System

As opposed to vertebrates, insects do not have a developed acquired immunity. Instead, they have a robust innate immunity that can be subclassified into cellular and humoral responses [88]. Cellular immunity is characterized by the action of defense cells (hemocytes) in aggregation, phagocytosis and encapsulation processes. The humoral immune response comprises the activation of antimicrobial peptides, of reactive oxygen species (ROS), and of enzyme complexes that regulate melanization and the coagulation cascade, among them the phenoloxidase (PO) [93,94].

In this context, previous data from our group indicated that JBU and the ubiquitous Soybean Urease (uSBU) are able to induce activation of the insect immune response in *R. prolixus* [48,95]. Defferrari and coworkers demonstrated that JBU is capable of activating both, the cellular and humoral immune responses. The activation of cellular aggregation induced by JBU is mediated by the cyclooxygenase (COX) pathway and required extracellular Ca^++^ ions. JBU also elicited the darkening of the hemolymph, an immune response associated with the melanization reaction triggered by the PO. At cellular level, immunolocalization assays demonstrated that the toxin is capable of inducing cytoskeleton damage and nuclear condensation in hemocytes [95]. Additionally, Martinelli and collaborators reported that uSBU in vivo and in vitro is also capable of inducing hemocyte aggregation in *R. prolixus* [48].

Based on these studies, cellular and biochemical approaches were carried out in order to evaluate the effects of Jaburetox and Soyuretox on the immune response of *R. prolixus*. Like JBU and uSBU, Jaburetox and Soyuretox induced Ca^++^-dependent aggregation of hemocytes in vivo and in vitro, mediated by the COX pathway [64,82]. Despite the aggregation, Fruttero et al. and Moyetta et al. demonstrated that the phagocytic capacity of hemocytes is not altered by the toxin [77,82]. In addition, Jaburetox also generated chromatin condensation, cytoskeleton disorganization and caspase 3 activation in the hemocytes, indicating the induction of apoptosis by the toxin [82]. The interaction of Jaburetox with the hemolymphatic cells was also seen upon in vivo and in vitro treatments, and the peptide was found in different subcellular locations [77].

Besides affecting the cellular immune response, Jaburetox also modulates the humoral immunity. In *R. prolixus*, the toxin induced an increment in the PO activity in vivo, without altering the activity of other effectors, such as the antibacterial cecropins and lysozymes [82]. Jaburetox triggered in hemocytes the increment of NOS gene expression. NO produced by the enzyme is known to induce the formation of free radicals that aid in immune defenses. However, these changes in gene expression were not accompanied by the corresponding modifications in protein levels in hemocytes or in enzymatic activity of NOS assayed in vitro, after the exposure to the toxin [77,82]. Through fluorescence assays with specific probes, it was observed that cells aggregated in the presence of Jaburetox had a greater local production of NO [77,82]. In 2020, Grahl et al. demonstrated in cultured hemocytes that a high dose of Jaburetox (6 µM) induced a significant increase of ROS production without altering cell viability [83].

When the Jaburetox-treated insects were injected with the pathogenic bacterium *Staphylococcus aureus*, the bacterial clearance was significantly reduced, indicating an immunosuppressive effect. Thus, the cellular and humoral immune activations triggered by Jaburetox do not protect the insect against posterior bacterial challenges [82]. These responses are similar to those elicited by bacterial and protozoan pathogens, raising the possibility that Jaburetox is recognized by the innate insect immunity as a pathogen-associated molecular pattern.

Another important immune response is the release of extracellular nucleic acid traps [96]. This immune mechanism of vertebrates and invertebrates is characterized by ROS-dependent release of chromatin into the cytoplasm, promoting the association of the nuclear material with antimicrobial proteins. Thereafter, this complex is released to the extracellular medium to withstand infections [96,97]. In this context, considering the changes in gene expression and nuclear condensation induced by Jaburetox in *R. prolixus*, experiments were designed to evaluate the impact of Jaburetox on the interactions of nucleic acids (DNA and RNA) extracted from the same insect species and used to mimic extracellular nucleic acid traps. It was observed that injection of the toxin together with RNA caused an increase in hemocyte aggregation, however when the toxin is injected together with DNA, no aggregation was seen. Concerning humoral responses, Jaburetox plus RNA yielded an increased PO activity only 6 h after injection, while Jaburetox plus DNA sustained an augmented humoral response both at 6 and 18 h after injection [83].

The effect of extracellular nucleic acids on the Jaburetox-induced immunosuppressive effect against pathogenic bacteria was also studied. Immunocompetence assays injecting Jaburetox alone, or Jaburetox plus DNA or RNA before the injection of bacteria, demonstrated that both RNA and DNA counteracted Jaburetox effects, and restored the bacterial-clearance capacity of the insects [83].

**Figure 3:**
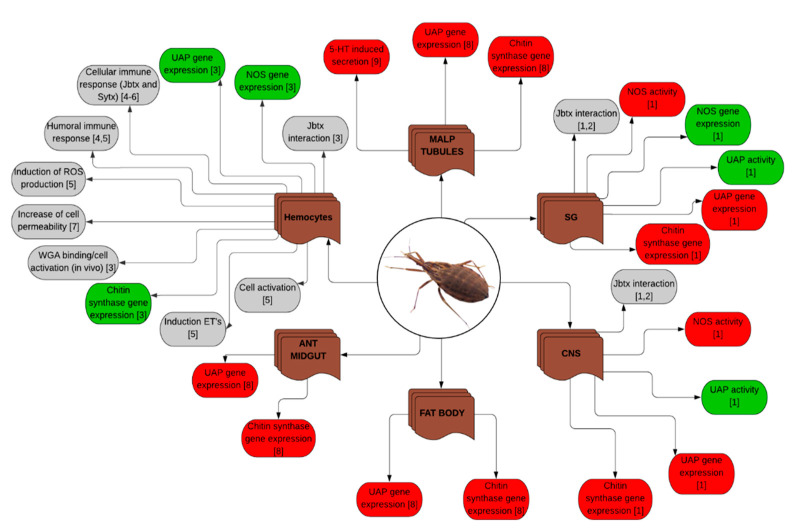
Tissue-specific effects of urease-derived peptides in *Rhodnius prolixus*. In the flowchart, the brown boxes are the organs or the cells affected by the urease-derived peptides while the grey boxes indicate no change. The green boxes represent an increase in the assessed effect while the red boxes indicate a decrease. Acronyms stand for: 5-HT, Serotonin; ANT MIDGUT, Anterior midgut; CNS, Central nervous system; ET’S, Extracellular traps; JBTX, Jaburetox; MALP TUBULES, Malpighian tubules; NOS, Nitric oxide synthase; ROS, Reactive oxygen species; SG, Salivary glands; SYTX, Soyuretox; UAP, UDP-*N*-acetylglucosamine pyrophosphorylase; WGA, Wheat germ agglutinin. The numbers between brackets indicate the corresponding references: (1) Fruttero et al., [78]; (2) unpublished results; (3) Moyetta et al., [77]; (4) Fruttero et al., [82]; (5) Coste Grahl et al., [83]; (6) Kappaun et al., [64]; (7) and (8) unpublished results; (9) Staniscuaski et al., [81].

Finally, to better understand the immunological modulation caused by nucleic acids, the toxin ability to induce the release of extracellular nucleic acid traps was evaluated. It was seen that Jaburetox was not able to induce the release of RNA or DNA, either upon in vitro or in vivo treatments. The incapacity of the insects to release extracellular nucleic acids after Jaburetox treatment could partly explain the immunosuppressive effect of the peptide and the weakened response of the treated insects against a bacterial challenge [83]. Since *R. prolixus* has been an instrumental model to understand the effects of the urease-derived peptides, we have summarized all our findings in the Figure 3.

### 4.2. Antifungal and Antibacterial Activity

So far there are not many examples in the literature of IDPs with antifungal activity, despite the two decades elapsed since the definition of IDPs, around the 2000s [98,99,100,101].

As mentioned above, Jaburetox presents antifungal activity against filamentous fungi and yeasts [61]. Postal and co-authors observed the toxicity of Jaburetox against the phytopathogenic filamentous fungi *Mucor* sp. (at 10 µM) and *Penicillium herguei* (at 20 µM). *Rhizoctonia solani* was not susceptible to Jaburetox in the tested doses. Regarding yeasts, Jaburetox at 9 µM inhibited the multiplication of *Saccharomyces cerevisiae*, *Candida parapsilosis* and *Pichia membranifaciens* and at 18 µM, the peptide inhibited *Candida tropicalis*, *C. albicans* and *Kluyveromyces marxiannus*. Fluorescence microscopy of *S. cerevisiae* evidenced an increase in membrane permeability in Jaburetox-treated cells, using the SYTOX Green stain. In *C. tropicalis,* exposition to Jaburetox also induced the formation of pseudohyphae. These microscopy experiments were conducted at lower doses of Jaburetox (0.36–0.72 µM). In another work of our group, Broll and co-workers [62] showed that FITC-labeled Jaburetox interacted with *S. cerevisiae* cells, and remained bound to membrane cell debris even after yeast lysis. These results suggested that the target of Jaburetox is present on the yeast external membrane [62].

The antimicrobial activity of Jaburetox against some bacteria such as *Bacillus cereus, Escherichia coli, Pseudomonas aeruginosa* and *Staphylococcus aureus* was observed in preliminary assays in the dose range of 0.25 μM to 13.5 μM [60]. As described earlier, Jaburetox was shown to permeabilize model membranes, as LUVs and PLBs, composed by different phospholipids and net charges; phosphatidylglycerol (PG) and phosphatidic acid (PA) with negative charges and the neutral phosphatidylethanolamine (PE), phosphatidylcholine (PC) and cholesterol (Ch) [66,76]. Many microorganisms contain negatively charged lipids in their membrane compositions, as PG and cardiolipin (CL) [102,103]. The main phospholipids found in the bacterium *S. aureus* membrane are PG, CL, and lysophosphatidylglycerol (LPG) [104,105]. In yeasts, a study using eight *C. albicans* azole-resistant and azole-sensitive strains demonstrated that the major phospholipids compositions in the plasma membrane of all the isolates were PC, PE, phosphatidylinositol (PI) and phosphatidylserine (PS). The percentage of phospholipids varied individually [106]. Interestingly, both microorganisms are susceptible to Jaburetox [60,61].

Soyuretox was also investigated regarding its antifungal activity. It was found to be active against *C. albicans*, *C. parapsilosis* and *S. cerevisiae* at 9 µM and 18 µM concentrations, similar to the fungitoxic doses reported for Jaburetox. For *C. albicans*, at the minimal inhibitory of 5 µM, production of superoxide anions was detected as part of the fungitoxic mode of action of Soyuretox. Binding of Soyuretox to *C. albicans* cells was observed by immunofluorescence [64].

**Figure 4:**
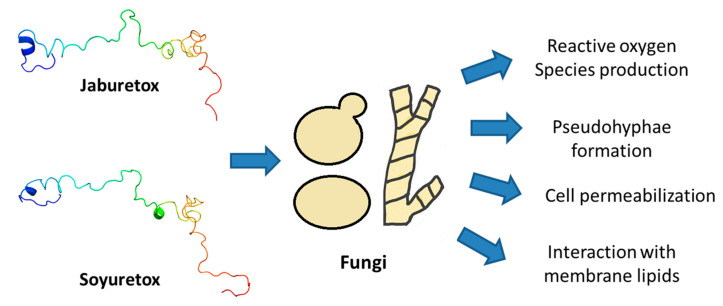
Schematic representation of the antifungal effects of Jaburetox and Soyuretox against filamentous fungi and yeasts.

The mechanism of antifungal activity of Jaburetox and Soyuretox remains elusive. It is known that the peptides permeabilize the fungal membrane, and cause change of fungal morphology, inducing formation of pseudohyphae, structures considered a stress and defense response mechanism of yeasts [48]. Moreover, the peptides induced intracellular production of superoxide anions in yeasts, causing oxidative stress. Our data suggest that these peptides probably interact with lipids in the fungal membrane (Figure 4). Although it still lacks experimental demonstration, it is plausible that the IDP nature of these peptides could be relevant for their antimicrobial activities, as changes in order-disorder states upon ligand binding could possibly modulate their fungitoxic action.

Jaburetox and Soyuretox are peptides prone to aggregation. In the studies aiming to characterize their 3D structures, TCEP, a potent reducing agent, was used to avoid aggregation during NMR experiments. It was demonstrated that Jaburetox and its truncated peptides (jbtx N-ter and jbtx C-ter) tend to form aggregates in solution and that their oligomerization state interfered in biological activities and membrane interactions [58,66,76]. Aggregation is known as an important factor in mode of action of antimicrobial peptides [102].

## 5. Structural Aspects of Other Intrinsically Disordered Bioactive Polypeptides

Even though approximately 30% of eukaryote proteins have disordered regions composed of fifty or more amino acid residues [99], there are relatively few reports of antimicrobial and insecticidal IDPs in the literature. Without aiming to be exhaustive, we briefly discuss here some examples and establish comparisons to Jaburetox and Soyuretox when relevant.

Plants are an inexhaustible source of bioactive molecules, including those that are part of their highly evolved defense mechanisms [34]. One important example are the cyclotides, naturally occurring macrocyclic peptides found in several families of plants. They present a unique head-to-tail cyclized backbone, stabilized by three disulfide bonds forming a cystine knot. This arrangement makes cyclotides exceptionally stable against chemical, thermal and biological degradation. These macromolecules are able to cross cellular membranes and control intracellular protein-protein interactions, enabling them to act upon different targets [107]. Cyclotides present diverse host-defense roles including insecticidal activity and it is believed that this property is derived from their ability to bind to membranes and form pores [108]. Kalata B1 is the most studied cyclotide, derived from the African plant *Oldelandia affinis* [109,110]. Daly et al. [111] reported that the N-terminal pro-domain of the kalata B1 precursor is intrinsically unstructured. This terminal region induces the self-association of the precursor to form a dimeric structure, which can, in turn, be determinant for the role of the N-terminal as a vacuolar-targeting signal. According to the authors, the disorder in the terminal region could be linked either to the fact that it is a functional segment with higher mobility or because it partially folds upon binding to a target, as could be also the case for Jaburetox and Soyuretox [64,69] Thus, the pore-forming capacity seems to be part of the toxic mechanism of both, urease-derived peptides and cyclotides. However, the intrinsically disordered nature of the cyclotides does not seem to be related to their insecticidal effect but rather to a role in signaling. The relevance of IDPs in signal transduction in plants is better documented and, in this case, it is believed that the intrinsically disordered nature is necessary to confer the low affinity and high specificity needed to perform the required interactions [112].

Concerning antifungal IDPs, histatins are a family of small, histidine-rich, cationic proteins present in mammalian saliva that constitute the first line of defense against oral candidiasis caused by *C. albicans* and to other pathogenic fungi. Histatin 5, an intrinsically disordered model protein, is the major histatin component of the unstimulated parotid secretion and the most potent antifungal protein of all the histatin family [113,114]. Histatin-5 has antifungal activity against *C. albicans* at 15 µM [115], a similar fungitoxic concentration for Jaburetox and Soyuretox [61,64]. The physiological concentration of histatin-5 in human saliva is 15 to 50 µM, while the concentration of protein required to kill half of maximum number of cells (ED_50_) is 1.4 µM. There is an extensive debate regarding the mode of action of this protein, with evidences pointing against pore formation or membrane lysis. The targets of histatin-5 appear to be intracellular and, once taken up by cells, it affects mitochondrial functions causing oxidative stress and ultimately killing the cells by ion imbalance and volume dysregulation induced by osmotic stress [116]. In addition, this peptide is related to depletion of intracellular ATP content and also oxidative damage due to ROS formation in intracellular organelles [117]. The production of oxidative molecules by histatin-5 is a common aspect with the mode of actions of Jaburetox and Soyuretox, which induced ROS generation both in insect hemocytes [83] and in *C. albicans* cells [64]. Since the fungitoxic mode of action of histatin-5 is not completely understood, it is not clear how the intrinsically disordered nature of the protein participates in the process. Nevertheless, histatin-5 mechanism of action against *C. albicans* is similar to what is known so far for Jaburetox and Soyuretox, including membrane interaction and permeabilization, and ROS formation. There is also evidence that Jaburetox is taken up by hemocytes [77], thus suggesting intracellular targets.

Hornerin is an IDP of 254 kDa that belongs to the S100-fused-type family. This protein is believed to be one of the main reasons why healthy human skin is remarkably resistant towards the infection by *Pseudomonas aeruginosa*, an environmental opportunistic pathogen widespread in water and soil [118]. Recently, fragments of hornerin were characterized as potent microbicidal agents and that this feature is maintained, independent of the amino acid sequence, provided they are linear cationic peptides containing a high percentage of disorder-promoting amino acids and a low percentage of order-promoting ones. The authors reported that the antimicrobial capacity of these cationic intrinsically disordered antimicrobial peptides (CIDAMPs) depends on their chain length, net charge, lipidation and environmental conditions [119]. The CIDAMPs have an intracellular mode of action, as hornerin transverses bacterial membranes by an energy-dependent mechanism and accumulates in the cytoplasm. The molecular targets of CIDAMPs seems to be different sites of the protein synthesis machinery [120]. The described features of CIDAMPS and the other IDP active peptides are summarized in Table 3.

In future works we intend to evaluate Jaburetox-derived peptides, its N- and C-termini portions as generated by Martinelli and co-authors [58] against fungi, in order to identify the fungitoxic region of the molecule. There are differences in secondary structure of the two terminal regions, the N-terminus being more disordered than the C-terminus [69] and this difference could be important to the antifungal activity. In addition, the C-terminal domain of Jaburetox interacts more effectively with lipid membranes [58]. More studies are required to answer these questions.

## 6. Biotechnological Applications and Perspectives

The use of GM crops resistant to pests such as fungi, nematodes and insects is an appealing strategy considering the current need of efficiently increasing the yield of the agricultural production with less impact in the environment and health [121]. Since their discovery in the 80′s, the use of transgenic crops has been dominated by the Bt technology. Nevertheless, some insect species are not susceptible to them and its intensive application has led to the development of resistance [19,122]. Considering the fact that IDPs do not need to fold in a proper way to be biologically active, this feature can potentially be an advantage regarding their expression in transgenic plants. In the case of Jaburetox, its disordered structure gives the peptide the capacity to withstand a vast range of temperatures and pH without losing its biological activity [69], a desirable feature for a biotechnological tool. Moreover, the conformational flexibility of Jaburetox and Soyuretox allows them to interact with several binding partners with different subcellular distributions, leading ultimately to diverse targets. This feature gives them the ability of avoiding or at least delaying the generation of resistance.

In this context, three types of transgenic crops expressing urease-derived peptides have been developed with promising results [87,88,123]. Soybean plants overexpressing Soyuretox were challenged with the root-knot nematode *Meloidogyne javanica*, a major agricultural pest in several countries [123]. As a result, the average reproductive factor of the nematode was significantly reduced. On the other hand, Didoné [87] reported that maize (*Zea mays*) expressing Jaburetox fed to the important polyphagous pest *S. frugiperda* led not only to a 39% lethality of larvae, but also to other sub-lethal statistically significant effects, such as body weight reduction, decreased ingestion and remarkably, fertility decline. In addition, Ceccon [88] demonstrated that Jaburetox-expressing tobacco plants also produced high mortality and a pronounced reduction in the leaf consumption by the lepidopteran *H. armigera.* These authors started the development of gene stacking strategy, with plants expressing simultaneously Jaburetox and a double-stranded RNA complementary to the *rieske* gene, which has the advantage of diminishing the possibility of resistance development events [87]. The use of plants overexpressing Soyuretox or Jaburetox would, in principle, be a way to avoid harming beneficial or innocuous insects, since only those species that fed on the plants would be affected. Nevertheless, since off-target effects could be an issue related to the broad insecticidal activity of Jaburetox/Soyuretox, transgenic crops can be improved using tissue-specific or damage-induced promoters [124,125].

These latest studies [87,88,123] are a proof of concept that IDPs in general, and the urease-derived peptides in particular, are very attractive pesticides that can be engineered for use as an effective and environmental-friendly strategy, alone or in combination with other IDPs or toxic molecules. The multidisciplinary research approaches employed by our group and collaborators improved significantly the understanding of the structural and biological aspects of these IDPs and encourage us to pursue a full comprehension of their mechanism of action that would, ultimately, facilitate their application in the field.

## Figures and Tables

**Table 1:** Compiled data from structural studies of Jaburetox and Soyuretox.

Approach	Peptide(s)	Data Obtained	Reference
*Ab initio* modeling	Jaburetox	A β-hairpin motif was observed in the C-terminal region of the molecule. It was hypothesized that the β-hairpin motif could be involved in the entomotoxic activity.	[57,66]
Molecular dynamics simulation (in aqueous system for 500 ns)	Jaburetox, its N- and C-termini peptides and Soyuretox	Jaburetox became more unstructured in its N-terminal portion, containing a few secondary structural elements and the major part of molecule in random coil. The β-hairpin structure was conserved in the C-terminal domain. The N-terminal peptide became totally unfolded and C-terminal showed a stabilization with β-sheet structures. Soyuretox became more globular in solution and showed changes in its secondary structure, with loss of helices and beta strands.	[58,64]
Dynamic Light Scattering and Small Angle X-ray Scattering Dynamic Light Scattering	Jaburetox Jaburetox	Demonstrated the ability of Jaburetox to interact with lipids using platelet-like multilamellar liposomes (PML). Jaburetox in a neutral solution is found in a single oligomeric form, exhibiting a large hydrodynamic radius, suggestive of a disordered polypeptide.	[67] [69]
Circular dichroism (CD) spectroscopy	Jaburetox and Soyuretox	Jaburetox showed a typical random coil conformation and small amount of secondary structure under native state. Jaburetox increased its secondary structure content when in contact with SDS-micelles and large unilamellar vesicles (LUVs) composed by phospholipids of different net charges. Jaburetox and Soyuretox showed disordered behavior at pH 6.5. Soyuretox acquired some secondary structure at pH 8.	[69] [62] [64]
Nuclear Magnetic Resonance (NMR) spectroscopy	Jaburetox Soyuretox	The heteronuclear single quantum coherence (HSQC) spectrum unveiled low signal dispersion in the proton dimension; the SSP analysis of chemical shifts predicted that Jaburetox is widely disordered with a small tendency to form α-structures. The 3D structure obtained from nuclear Overhauser enhancement (NOE) do not demonstrated the presence of a stable tertiary structure. The HSQC NMR spectrum obtained for Soyuretox showed a low signal dispersion in the proton dimension. A more ordered structure of Soyuretox in the presence of SDS micelles (10 mM) was also confirmed in the peptide’s HSQC NMR spectrum, demonstrating a widening of signal dispersion.	[69] [64]

**Table 2:** Effects of Jaburetox and Soyuretox in different species of insects.

Species	Stage(s)	Assay	Toxic Peptide(s)	Effect(s)	Reference
*Dysdercus peruvianus*	Nymphs	Feeding	Jaburetox	Lethality	[57,80]
	Nymphs	Feeding and injection	Soyuretox	Lethality	[64]
*Oncopeltus fasciatus*	Nymphs	Injection	Jaburetox	Lethality	[58]
	Nymphs	Injection, feeding	Jaburetox	Lethality	[58,80]
*Rhodnius prolixus*	Nymphs and adults	Injection, feeding, in vitro	Jaburetox and Soyuretox	Effects on diuresis, enzymatic activities, expression of genes, cell activation and immune response, interaction of Jaburetox with the central nervous system and the salivary glands, among others (see Figure 3)	[58,64,77,78,81,82,83], unpublished results
	Nymphs and adults	Injection	Jaburetox	Lethality	[80]
*Triatoma infestans*	Adults	Injection	Jaburetox	Lethality, behavioral alterations, neurotoxicity, localization of the peptide in the central nervous system, interaction of the peptide with UDP-*N*-acetylglucosamine pyrophosphorylase (UAP), inhibition of nitric oxide synthase (NOS) activity	[84]
*Phoetalia pallida*	Adults	Injection	Jaburetox	Blockade of evoked contractions of coxal muscle	[58]
		In vitro	Jaburetox	Interaction of the peptide with the central nervous system	[62]
*Nauphoeta cinerea*	Adults	Injection	Jaburetox	Alteration of locomotor behavior, leg and antennae grooming, neuromuscular blockade, cardiotoxicity and alterations in nerve and muscle electrophysiological profiles	[85]
	Adults	Feeding, injection, in vitro	Jaburetox	Absence of lethality, modulation of NOS, UAP and acetylcholinesterase activity in the central nervous system	[86]
*Blatella germanica*	Nymphs	Feeding	Jaburetox	Lethality	[57]
	Larvae	Feeding	Jaburetox	Lethality and weight reduction	[57]
*Spodoptera frugiperda*	Larvae	Feeding on transgenic corn plants	Jaburetox	Weight reduction, reduced feed consumption, sterility of females and lethality	[87]
	Larvae	Feeding	Jaburetox	Lethality and delay in larval development	[87]
*Helicoverpa armigera*	Larvae	Feeding on transgenic tobacco plants	Jaburetox	Lethality and reduced feed consumption	[88]
*Aedes aegypti*	Larvae	Feeding	Jaburetox	Lethality	[60]

**Table 3:** Other intrinsically disordered proteins with biological activities.

IDP	Source	Biological Activity	Disorder Region	Reference
Kalata B1	African plant *Oldelandia affinis*	Signaling, ability to bind and to form pores in membranes	N-terminal pro-domain	[111]
Histatin 5	Mammalian saliva	Antifungal activity	No defined structure in solution	[116,117]
Hornerin	Human skin	Antibacterial activity	Almost all the protein is unstructured, except the N-terminus. Cationic peptides generated from hornerin present antimicrobial activity	[119]
